# Single-cell transcriptomes reveal the mechanism for a breast cancer prognostic gene panel

**DOI:** 10.18632/oncotarget.26044

**Published:** 2018-09-07

**Authors:** Shengwen Calvin Li, Andres Stucky, Xuelian Chen, Mustafa H. Kabeer, William G. Loudon, Ashley S. Plant, Lilibeth Torno, Chaitali S. Nangia, Jin Cai, Gang Zhang, Jiang F. Zhong

**Affiliations:** ^1^ Neuro-oncology and Stem Cell Research Laboratory, CHOC Children's Research Institute, Children's Hospital of Orange County; Department of Neurology, University of California-Irvine School of Medicine, Orange, CA, USA; ^2^ Division of Periodontology, Diagnostic Sciences and Dental Hygiene, and Division of Biomedical Sciences, Herman Ostrow School of Dentistry, University of Southern California, Los Angeles, CA, USA; ^3^ Pediatric Surgery, CHOC Children's Hospital, Department of Surgery, University of California-Irvine School of Medicine, Orange, CA, USA; ^4^ Neuroscience Institute, Children's Hospital of Orange County (CHOC), Gamma Knife Center of Southern California, Department of Neurosurgery, University of California-Irvine School of Medicine, Orange, CA, USA; ^5^ Division of Pediatric Oncology, Children's Hospital of Orange County, Orange, CA, USA; ^6^ Hyundai Cancer Institute at CHOC Children's Hospital, Oncology, Bone and Soft Tissue Tumor Program, After Cancer Treatment Survivorship Program, CHOC Children's Hospital, Orange, CA, USA; ^7^ Chan Soon-Shiong Institute for Medicine, Verity Medical Foundation, Laguna Hills, CA, USA; ^8^ Department of Oral and Maxillofacial Surgery, Zhuhai People's Hospital, Zhuhai, China; ^9^ Department of Oral and Maxillofacial Surgery, Xinqiao Hospital, Army Medical University, Chongqing, China

**Keywords:** cell cycle, single-cell, cell-cycle-staged therapy, transcriptome, cell cycle phase

## Abstract

The clinical benefits of the MammaPrint^®^ signature for breast cancer is well documented; however, how these genes are related to cell cycle perturbation have not been well determined. Our single-cell transcriptome mapping (algorithm) provides details into the fine perturbation of all individual genes during a cell cycle, providing a view of the cell-cycle-phase specific landscape of any given human genes. Specifically, we identified that 38 out of the 70 (54%) MammaPrint^®^ signature genes are perturbated to a specific phase of the cell cycle. The MammaPrint^®^ signature panel derived its clinical prognosis power from measuring the cell cycle activity of specific breast cancer samples. Such cell cycle phase index of the MammaPrint^®^ signature suggested that measurement of the cell cycle index from tumors could be developed into a prognosis tool for various types of cancer beyond breast cancer, potentially improving therapy through targeting a specific phase of the cell cycle of cancer cells.

## INTRODUCTION

Breast cancer remains one of the most devastating diseases worldwide. Traditionally, estrogen receptor (ER), progesterone receptor (PR) and Her2 have been used to classify breast cancer into three subtypes: positive for hormonal receptors (ER+), Her2+, and triple negative (TNBC) without these receptors. While ER+ tumors are treated with selective ER modulators or aromatase inhibitors and Her2+ tumors are treated with antibodies against this receptor [1], TNBC tumors do not have a specific treatment, prompting the search for innovative approaches. A genome-wide association study (GWAS) revealed the tumor necrosis factor superfamily member 13B (*TNFSF13B*) gene, suggesting new target-specific therapies [2]. Translation of these transcriptome results to clinical practice is underway. The clinical significance of a breast-cancer-associated, 70-gene signature panel (a.k.a., MammaPrint^®^ signature) was first reported as a clinical tool to help refine prognosis in the NEJM fourteen years ago [2], as implemented by Agendia BV, Amsterdam, The Netherlands for scale-up clinical trials. Initially, van ‘t Veer and colleagues screened a total of 25,000 genes of using microarray technology for supervised clustering on mRNA expression of 70 genes and van ‘t Veer *et al*. validated for either a low or high risk of patients. The 70-genes assay was adopted in the US for clinical practice since van ‘t Veer moved to University of California, San Francisco. Based on the 70-gene MammaPrint^®^ signature, the same group reported a follow-up *NEJM* article, showing that “no chemotherapy led to a 5-year rate of survival without distant metastasis that was 1.5% lower than the rate with chemotherapy”, with 1550 patients (23.2%) at high clinical risk and low genomic risk for recurrence, out of a randomized Phase 3 study with 6693 enrolled early-stage breast cancer patients [3]. This suggests that approximately 46% of women at high clinical risk may not need chemotherapy. Monitoring the MammaPrint^®^ 70-gene signature can guide the treatment. However, these genes were selected empirically from breast cancer cases through time. It is not clear why these genes have predictive power and whether such a panel can be applied to other types of cancers.

Here, we report a new algorithm to cluster genes that share the same cell cycle phase (i.e., G_0_, G_1_, S, or G_2_) based on a spectrum of single-cell transcriptomes from a cell-cycle model system. This algorithm allows cells to be sorted into subpopulations of sharing the same cell-cycle phases. We inferred a possible mechanism by which predictive power of MammaPrint^®^ signature predicts its clinical outcomes for breast cancer.

## RESULTS

**Figure 1:**
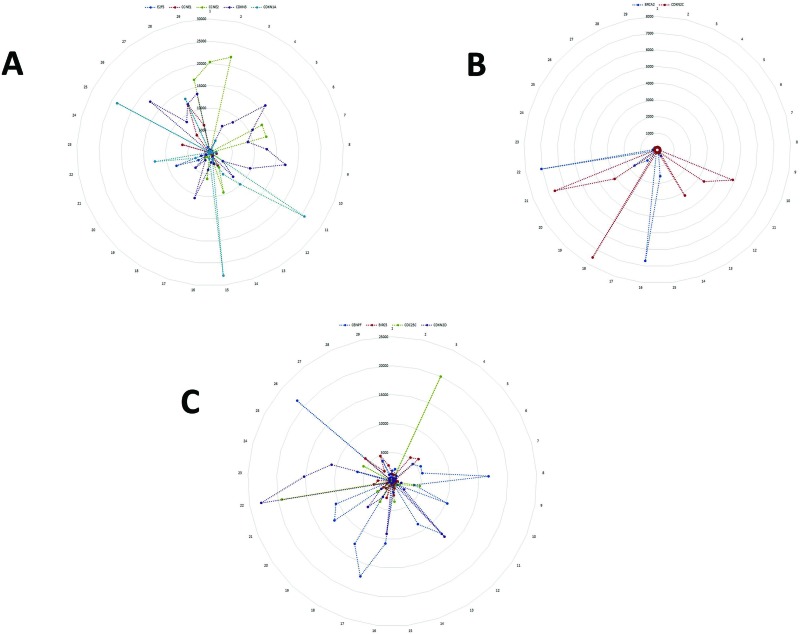
Sequential perturbations of cell-cycle-specific genes in a single-cell model system After organizing single-cell transcriptomes by similarity into a sequencing order, expression levels of various cell-cycle-specific genes were plotted to visualize the sequential perturbation of individual genes during the cell cycle. Cell cycle phases were defined and colored based on the cell cycle molecular map. As expected, G0/G1-specific genes had higher expression levels in the G0/G1 phase (**A**) and G2/M-specific genes had high expression levels in the G2/M phase (**B**). G2/M-specific genes had high expression levels in the G2/M phase and the early G0/G1 phase (**C**). Note: the numbers along the outside circle (#1 – 29) represent the cell cycle phase: #1- #15 for G1-phase; #16-#22, S-phase; #23-#29, G2/M-phase. The number on the vertical scale radiating from the center represents the level of gene expression with the center representing 0, the lowest, scaling up to the outer circle, the highest.

We defined phase-specific, cell-cycle-dependent single-cell transcriptomes using the model system - Fucci cells, which have fluorescent cell-cycle phase-specific indicators. We obtained single-cell transcriptomes from these Fucci cells with our microfluidic platform with nanoliter reactors [5]. Combining these two technologies allowed for the characterization of a cell cycle phase-specific map using a similarity matrix (algorithm) based on known cell cycle genes (GO:0022402). We used this algorithm to create a novel cell cycle map of known cell cycle genes in the corresponding sequential order (Figure 1). As expected, known cell cycle genes had expression perturbation profiles that agreed with previously reported studies of physical cell lysates. In addition to known cell cycle genes, genes indicated by the Self-Organizing Map (SOM) analysis were also plotted onto the cell cycle map to identify novel candidate cell cycle genes, termed cell cycle index.

**Figure 2:**
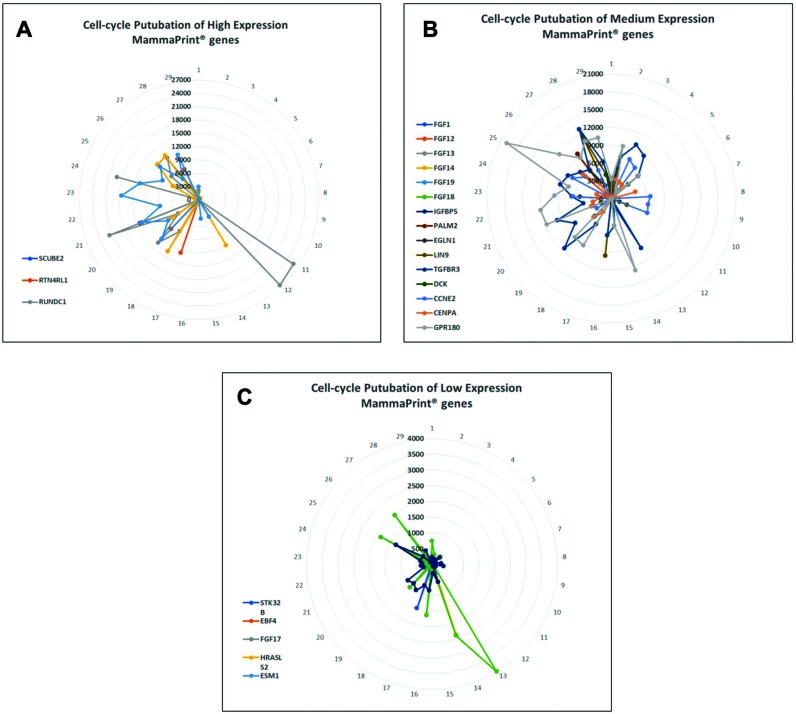
Perturbation of MammaPrint^®^ genes during cell cycle suggests that many MammaPrint^®^ genes are cell cycle regulators With microfluidic devices, transcriptomes of individual cells were arranged by similarity to construct a cell cycle map with 29 single-cells with each single-cell represented a specific stage of the cell cycle. The distance between cells represent their similarity with neighboring cells. The map reveals the stepwise perturbations of all genes during the cell cycle, such as G1-phase, S-phase, and G2-phase. The mRNA perturbation of majority of MammaPrint^®^ genes was plotted and presented by expression levels. (**A**) Highly expression MammaPrint^®^ gene; (**B**) medium expression MammaPrint^®^ genes and (**C**) low expression MammaPrint^®^ genes. Genes at all level of expression showed cell-cycle dependent perturbation patterns. These results suggest that majority of MammaPrint^®^ genes are cell cycle regulators and MammaPrint^®^ gene panel is a cell cycle index panel.

We applied this algorithm to assess the cell cycle activity of the MammaPrint^®^ 70-gene signature [4] to create a cell-cycle index for cell-cycle-phase-specific mapping as generated from single-cell transcriptomes. In addition to the previously reported 15 cell cycle-related genes [5, 6], our strategy revealed 23 additional cell cycle-associated genes among the 70 MammaPrint^®^ genes. Among the 23 newly identified cell cycle-related genes, we identified 15 genes regulating G1 phase (Figure 2B), 5 genes regulating S-phase (Figure 2C), and 3 genes regulating G2 phase (Figure 2A). More importantly, these cell cycle specific genes are associated with clinical outcomes, as judged with current database of breast cancer patients’ consequences in multiple reports and clinical trials, including cancer recurrence (Table 1), cancer pathological stage (Table 2), and primary versus metastatic disease (Table 3).

**Table 1:** Breast cancer recurrence governed by cell cycle regulated genes^*^

Gene name	Incidence of patient's recurrence	Expression median	Fold change	*P*-value	Gene rank	Reference
CCNE2	No Recurrence at 3 Years (*N* = 6)		−1.381	0.03	467 (in top 3%)	Esserman *et al*., 2012
	Recurrence at 3 Years (*N* = 3)		−0.654			Esserman *et al*., 2012
CENPA	1. No Recurrence at 3 Years (*N* = 10)	–0.44		1.76E-04	20 (in top 1%)	Desmedt *et al*., 2007
	2. Recurrence at 3 Years (*N* = 3)	1.197				
	1. No Recurrence at 5 Years (*N* = 9)	–0.707		2.29E-04	24 (in top 1%)	Desmedt *et al*., 2007
	2. Recurrence at 5 Years (*N* = 3)	1.197				
	1. Alive at 3 Years (*N* = 77)	–134		0.002	187 (in top 1%)	Esserman *et al*., 2012
	2. Dead at 3 Years (*N* = 15)	–0.355				
	1. Alive at 5 Years (*N* = 21)	–1.807		2.12E-04	26 (in top 1%)	Esserman *et al*., 2012
	2. Dead at 5 Years (*N* = 20)	–566				
	1. No Recurrence at 3 Years (*N* = 78)	1.373		0.01	380 (in top 2%)	Loi *et al*., 2007
	2. Recurrence at 3 Years (*N* = 8)	2.246				
	1. No Recurrence at 5 Years (*N* = 66)	–0.062		0.007	471 (in top 3%)	Loi *et al*., 2008
	2. Recurrence at 5 Years (*N* = 10)	0.604				
LIN9	No Recurrence at 3 Years (*N* = 69)	0.418		0.009	589 (in top 4%)	Esserman *et al*., 2012
	Recurrence at 3 Years (*N* = 24)	0.722				
	1. No Recurrence at 5 Years (*N* = 8)	0.594		0.011	924 (in top 5%)	Finak *et al*., 2008
	2. Recurrence at 5 Years (*N* = 11)	1.149				
RUNDC1		1.442		0.151	5579	TCGA Breast (database)
		–1.059		0.52	8666	Radvanyi *et al*., 2005
		–1.061		0.592	13627	Bittner (database)
BRCA2	Recurrence at 3 years (*N* = 4)	0.596		0.014	516 top 3%	Loi *et al*., 2008
	No Recurrence at 3 years (*N* = 72)	–0.076				Loi *et al*., 2008
	Recurrence or metastasis at 5 years (*N* = 6)	0.639		4.83E-04	87 top 1%	Loi *et al*., 2008
	No Recurrence or metastasis at 5 years (*N* = 69)	–0.09				Loi *et al*., 2008
	Recurrence at 5 Years (*N* = 10)	–0.257		0.043	861 top 6%	Ma *et al*., 2003
	No Recurrence at 5 Years (*N* = 22)	–1.602				Ma *et al*., 2003
	Recurrence at 3 Years (*N* = 3)	–1.427		0.038	958 top 8%	Desmedt *et al*., 2007
	No Recurrence at 3 Years (*N* = 10)	–2.141				Desmedt *et al*., 2007
CCNB1	Recurrence or metastasis at 3 years (*N* = 8)	3.124		0.002	103 top 1%	Loi *et al*., 2007
	No Recurrence or metastasis at 3 years (*N* = 78)	2.465				Loi *et al*., 2007
	Recurrence or metastasis at 5 years (*N* = 14)	3.124		0.023	1306 top 7%	Loi *et al*., 2007
	No Recurrence or metastasis at 5 years (*N* = 68)	2.465				Loi *et al*., 2007
	No Recurrence at 3 Years (*N* = 71)	–0.04		0.018	487 (in top 3%)	Loi *et al*., 2008
	Recurrence at 3 Years (*N* = 5)	0.853				Loi *et al*., 2008
	No Recurrence at 3 Years (*N* = 10)	1.338		0.046	1130 (in top 9%)	Desmedt *et al*., 2007
	Recurrence at 3 Years (*N* = 3)	2.709				Desmedt *et al*., 2008
CDC25A	No Recurrence at 1 Year (*N* = 269)	–1.985		0.002	312 (in top 3%)	Esserman *et al*., 2012
	Recurrence at 1 Year (*N* = 16)	–0.749				Esserman *et al*., 2012
	No Recurrence at 3 Years (*N* = 217)	–2.08		6.83E-04	532 (in top 5%)	Esserman *et al*., 2012
	Recurrence at 3 Years (*N* = 68)	–1.078				Esserman *et al*., 2012
	1. No Recurrence at 3 Years (*N* = 10)	–2.893		0.002	94 (in top 1%)	Esserman *et al*., 2012
	2. Recurrence at 3 Years (*N* = 3)	–0.037				Esserman *et al*., 2012
CDC25C	No Recurrence at 3 Years (*N* = 10)	–0.706		0.002	79 (in top 1%)	Loi *et al*., 2007
	Recurrence at 3 Years (*N* = 3)	–0.12				Loi *et al*., 2007
	No Recurrence at 5 Years (*N* = 9)	–0.879		0.002	82 (in top 1%)	Desmedt *et al*., 2007
	Recurrence at 5 Years (*N* = 3)	–0.12				Desmedt *et al*., 2007
	No Recurrence at 3 Years (*N* = 78)	0.193		0.006	245 (in top 2%)	Loi *et al*., 2007
	Recurrence at 3 Years (*N* = 8)	0.893				Loi *et al*., 2008
	No Recurrence at 5 Years (*N* = 68)	0.138		0.002	118 (in top 1%)	Loi *et al*., 2009
	Recurrence at 5 Years (*N* = 14)	0.893				Loi *et al*., 2010
	No Recurrence at 3 Years (*N* = 6)	–1.563		0.038	917 (in top 5%)	Esserman *et al*., 2012
	Recurrence at 3 Years (*N* = 5)	–0.795				Esserman *et al*., 2013
CDKN2D	No Recurrence at 5 Years (*N* = 68)	0.405		0.009	584 (in top 3%)	Loi *et al*., 2007
	Recurrence at 5 Years (*N* = 14)	0.6				
	No Recurrence at 3 Years (*N* = 229)	–0.051		0.001	491 (in top 4%)	van de Vijver *et al*., 2002 MammaPrint®
	Recurrence at 3 Years (*N* = 63)	0.01				MammaPrint 70-gene list
	No Recurrence at 5 Years (*N* = 68)	–0.059		9.53E-04	489 (in top 4%)	van de Vijver *et al*., 2002 MammaPrint®
	Recurrence at 5 Years (*N* = 14)	0.006				

^*^Novel phase-related genes in red on A column.

**Table 2:** Pathological stages of breast cancer governed by biomarkers expression^*^

Gene name	Stage and number of patients	Fold change	*P*-value	Gene rank	Reference
CCNE2	Grade 3 (10) vs Grade 2 (20)	2.073	0.002	149 (in top 1%)	MA XJ *et al*., 2004
CENPA	TP53 Mutation (84) vs TP53 Wild Type (557)	1.679	1.82E-16	1 (in top 1%)	Curtis *et al*., 2012
	ERBB2/ER/PR Negative (211) vs another Biomarker Status (1,340)	2.135	7.02E-57	25 (in top 1%)	Curtis *et al*., 2012
	Stage II (4) vs Stage I (3)	1.706	0.009	139 (in top 1%)	Curtis *et al*., 2012
	ERBB2/ER/PR Negative (39) vs Another Biomarker Status (129)	2.402	4.33E-08	94 (in top 1%)	Bittner *et al*., 2005
	M1+ (5) vs M0 (176)	2.327	1.00E-02	996 (in top 6%)	Bittner *et al*., 2005
	N1+ (12) vsN0 (7)	4.343	9.95E-05	12 (in top 1%)	Lu *et al*., 2008
	Grade 3 (4) vs Grade 2 (3)	2.221	8.00E-03	188 (in top 2%)	Desmedt *et al*., 2007
	TP53 Mutation (58) vsTP53 Wild Type (189)	1.67	3.94E-13	41 (in top 1%)	Ivshina *et al*., 2006
	TP53 Mutation (58) vsTP53 Wild Type (189)	1.502	5.17E-04	521 (in top 3%)	Gluck *et al*., 2012
	ERBB2/ER/PR Negative (39) vs other Biomarker Status (129)	2.086	5.80E-04	751 (in top 4%)	Richardson *et al*., 2006
	ERBB2/ER/PR Negative (39) vs other Biomarker Status (129)	3.625	5.90E-04	178 (in top 2%)	Zhao H *et al*., 2004
LIN9	N1+ (12) vs N0 (7)	1.658	7.86E-04	94 (in top 1%)	Lu *et al*., 2008
	ERBB2/ER/PR Negative (39) vs Another Biomarker Status (129)	1.627	1.00E-06	261 (in top 2%)	Bittner *et al*., 2005
	ERBB2/ER/PR Negative (18) vs Another Biomarker Status (19)	1.732	2.96E-04	575 (in top 3%)	Richardson *et al*., 2006
RUNDC1	Grade 3 (3) vs Grade 2 (24) vs Grade 1 (13)		0.038	1023 (in top 6%)	Curtis *et al*., 2012
	Grade 3 (17) vs Grade 2 (3)	1.183	0.044	3511 (in top 19%)	Nik-Zainal *et al*., 2012
BRCA2	Grade 3 (10) vs Grade 2 (20)	2.693	4.13E-06	4 (in top 1%)	MA XJ *et al*., 2004
	Dead at 1 Year (7) vs Alive at 1 Year (47)	1.962	3.10E-05	4 (in top 1%)	Sorlie *et al*, 2001
	Dead at 1 Year (12) vs Alive at 1 Year (69)	1.594	0.002	42 (in top 1%)	Sorlie *et al*, 2003
	Grade 3 (3) vs Grade 2 (7)	5.117	0.009	752 (in top 6%)	Desmedt *et al*., 2007
CCNB1	ERBB2/ER/PR Negative (39) versus other (129)	1.712	4.28E-05	811 top 5%	Bittner *et al*., 2005
	M0 (176) vs M1+ (5)	1.634	0.018	1461 top 8%	
	Grade 3 (10) vs Grade 2 (20)	1.782	0.006	299 top2 %	MA XJ *et al*., 2004
	N1+ (12) vs N0 (7)	3.198	3.56E-04	47 top 1%	Lu *et al*., 2008
CDC25A	Grade 3 (3) vs Grade 2 (7)	8.625	9.48E-04	175 (in top 2%)	Desmedt *et al*., 2007
	ERBB2/ER/PR Negative (39) vs positive	2.324	4.74E-05	838 (in top 5%)	Bittner *et al*., 2005
		2.452	3.04E-04	40 (in top 1%)	Ma XJ *et al*., 2004
CDC25C	Grade 3 (3) vs Grade 2 (7)	4.042	0.001	221 (in top 2%)	Desmedt *et al*., 2007
	Bloom-Richardson Grade 2 (8) vs Bloom-Richardson Grade 1 (5)	2.228	0.008	1130 (in top 6%)	Lu *et al*., 2008
	Elston Grade 3 (16) vs Elston Grade 2 (37) vs Elston Grade 1 (17)		1.04E-07	27 (in top 1%)	Loi *et al*., 2007
CDKN2D	M1+ (5) vs M0 (176)	1.921	7.76E-04	227 (in top 2%)	Bittner *et al*., 2005
	N1+ (16) vs N0 (14)	1.99	0.032	1455 (in top 8%)	Bittner *et al*., 2005
	Grade 3 (10) vs Grade 2 (20)	1.533	0.009	383 (in top 3%)	MA XJ *et al*., 2004
	M1+ (8) vsM1+ (8)	1.503	0.012	921 (in top 5%)	Kao KJ *et al*., 2011

^*^Novel phase-related genes in red on A column.

**Table 3:** Breast cancer biomarkers *In Silico* analysis: Metastasis vs primary^*^

Gene name	Metastasis vs primary	Fold change	*P*-value	Gene rank	Reference
CCNE2	Metastasis vs primary	1.681	0.058	2249	Bittner Breast database 2005
	Metastasis vs primary	1.214	0.307	3190	Weigelt *et al*., 2003
	Metastasis vs primary	–1.423	0.786	12226	Radvanyi *et al*., 2005
	Metastasis vs primary	–1.119	0.578	13819	TCGA 2005 database
CENPA	Metastasis vs primary	2.464	0.032	168	Weigelt *et al*., 2003
	Metastasis vs primary	1.057	0.131	2895	Haverty Breast
	Metastasis vs primary	1.521	0.096	3298	Bittner Breast database 2005
	Metastasis vs primary	1.828	0.155	4115	Radvanyi *et al*.., 2005
LIN9	Metastasis vs primary	1.445	0.174	5203	Bittner Breast database 2005
	Metastasis vs primary	–1.085	0.581	9471	Radvanyi *et al*., 2005
	Metastasis vs primary	–1.176	0.825	17493	TCGA 2005 database
RUNDC1	Metastasis vs primary	1.442	0.151	5579	TCGA 2005 database
	Metastasis vs primary	–1.059	0.52	8666	Radvanyi *et al*.., 2005
	Metastasis vs primary	–1.061	0.592	13627	Bittner Breast database 2005
BRCA2	Metastasis vs primary	–1.036	0.55	3551	Sorlie *et al*, 2003
	Metastasis vs primary	–1.034	0.61	6852	Weigelt *et al*., 2003
	Metastasis vs primary	1.09	0.326	8552	Bittner Breast database 2005
	Metastasis vs primary	–1.531	0.851	13239	Radvanyi *et al*., 2005
	Metastasis vs primary	–1.287	0.699	15588	TCGA 2005 database
CCNB1	Metastasis (9) versus primary (327)	1.506	0.05	2037 top 11%	Bittney database 2005
	Metastasis (5) versus primary (103)	–1.212	0.748	4538 top 74%	Sorlie *et al*., 2001
	Metastasis (6) versus primary (4)	–1.313	0.807	8893 top 87%	Weigelt *et al*., 2003
	Metastasis (7) versus primary (47)	–1.397	0.703	11075 top 67%	Radvanyi *et al*.., 2005
	Metastasis (3) versus primary (529)	–1.133	0.687	15399 top 76%	TCGA 2005 database
CDC25A	Metastasis vs primary	1.673	0.014	828	Bittner Breast database 2005
	Metastasis vs primary	–1.016	0.518	3417	Sorlie *et al*, 2003
	Metastasis vs primary	1.098	0.357	3809	Weigelt *et al*., 2003
	Metastasis vs primary	1.488	0.149	4018	Radvanyi *et al*.., 2005
	Metastasis vs primary	–1.407	0.76	16431	TCGA 2005 database
CDC25C	Metastasis vs primary	2.248	0.017	932	Bittner Breast database 2005
	Metastasis vs primary	1.101	0.1	1140	TCGA Breast 2 database
	Metastasis vs primary	1.618	0.082	593	Weigelt *et al*., 2003
CDKN2D	Metastasis vs primary	1.415	0.01	224	Sorlie *et al*, 2003
	Metastasis vs primary	1.464	0.535	3559	Bittner Breast database 2005
	Metastasis vs primary	–1.02		6008	Weigelt *et al*., 2003

^*^Novel phase-related genes in red on A column.

Specifically, nine genes of the 70 MammaPrint^®^ genes are regulated in a cell cycle specific pattern at cancer recurrence (Table 1), including CCNE2, CENPA, LIN9, RUNDC1, BRCA2, CCNB1, CDC25A, CDC25C, and CDKN2D. Four of these nine genes, CCNE2, CENPA, LIN9, RUNDC1, are newly defined as phase-related genes of cell cycle, which can be used as prognostic biomarkers for either 3- or 5-year survival with *p*-Value < 0.05. In particular, CCNB1 in the cohort of “Recurrence or metastasis at 3 years (*N* = 8 patients)” showed a 3.124-fold change (*p* = 0.002), in the cohort of “No Recurrence or metastasis” at 3 years (*N* = 78 patients)” had a 2.465-fold change, in the cohort of “Recurrence or metastasis at 5 years (*N* = 14 patients)” had a 3.124-fold change (*p* = 0.023), and in the cohort of “No Recurrence or metastasis at 5 years (*N* = 68 patients) had a 2.465-fold change (Table 1). In Table 3, CENPA *in Silico* analysis of breast cancer biomarkers for metastasis vs. primary showed a 2.46-fold change (*p*-Value = 0.031, while CDC25C, a 2.24-fold change, *p* = 0.017. In Table 2, biomarker-governed pathological stages consisted of these nine genes, in particular, CCNE2 showed a 2.073-fold change with *p* = 0.002; CENPA, associated ERBB2/ER/PR negative (211 patients) vs. other biomarker status (1,340 patients), a 2.402-fold change, *p* = 4.33E-08, and another cohort, CENPA associated with ERBB2/ER/PR negative (39 patients) vs. other biomarker status (129 patients), a 3.625-fold change, with *p* = 5.90E-04. In a small cohort, BRCA2-expression associated-Grade 3 (3 patients) vs. -Grade 2 (7 patients) showed a 5.117-fold change (*p* = 0.009), while CDC25A of Grade 3 (3 patients) vs. Grade 2 (7 patients) showed a 8.625-fold change (*p* = 9.48E-04) and CDC25A of ERBB2/ER/PR negative (39 patients) vs. positive had a 2.324-fold change with *p* = 4.74E-05. CDC25C in Grade 3 (3 patients) vs Grade 2 (7 patients) showed a 4.042-fold change (*p* = 0.001) while in the cohort of Bloom-Richardson Grade 2 (8 patients) vs. of Bloom-Richardson Grade 1 (5 patients), CDC25C had a 2.228-fold change with *p* = 0.008 (Table 2). In summary, 38 (more than half, 54%) of the 70 MammaPrint^®^ genes are involved in cell cycle control; nine of which are associated with breast cancer patients.

## DISCUSSION

Current commercially available assays include the MammaPrint^®^, OncotypeDX (the 21-gene Recurrence Score, with reverse transcriptase PCR (RT-PCR), PAM50/Prosigna (on the expression of 46 genes using quantitative PCR (qPCR), by NanoString Technologies, Seattle, WA), Endopredict (use the expression of 8 cancer-related and 3 reference genes determined with RT-PCR, by Myriad Genetics Inc, Salt Lake City, UT), of which four criteria is considered: “assay development and methodology, clinical validation, clinical utility and economic value” [7]. Although level IA clinical trial results show MammaPrint^®^ and OncotypeDX for prognostic information, clinical utility studies show a higher reduction in chemotherapy was achieved only by OncotypeDX. The inconsistency may be derived from inter-tumor and intra-tumor heterogeneity as these results were based on assays on bulky tumors. We have recently shown that a cancer relapse signaling pathway can be detected only in a single-cell transcriptome, not in a bulky tumor analysis, as bulky tumor transcriptomic analyses obscure the low signal of certain biomarkers [8]. We rationalized that single-cell transcriptomic analysis can enhance signal-to-noise ratio over bulky tumor transcriptomic analysis. Here, we discovered that a total of 38 genes (23 of them were revealed within the present work) measured with the MammaPrint^®^ assay are cell cycle regulated. We conclude that the MammaPrint^®^ assay would average out perturbations in gene expression when applied to a mixture of cells and thus remain undetected when using bulky tumors. We further illustrate that the advantage of our finding would make it possible (1) to attack the respective gene products using a combination therapy and (2) to deliver the right drugs to cells of the respective cell cycle stage.

**Figure 3:**
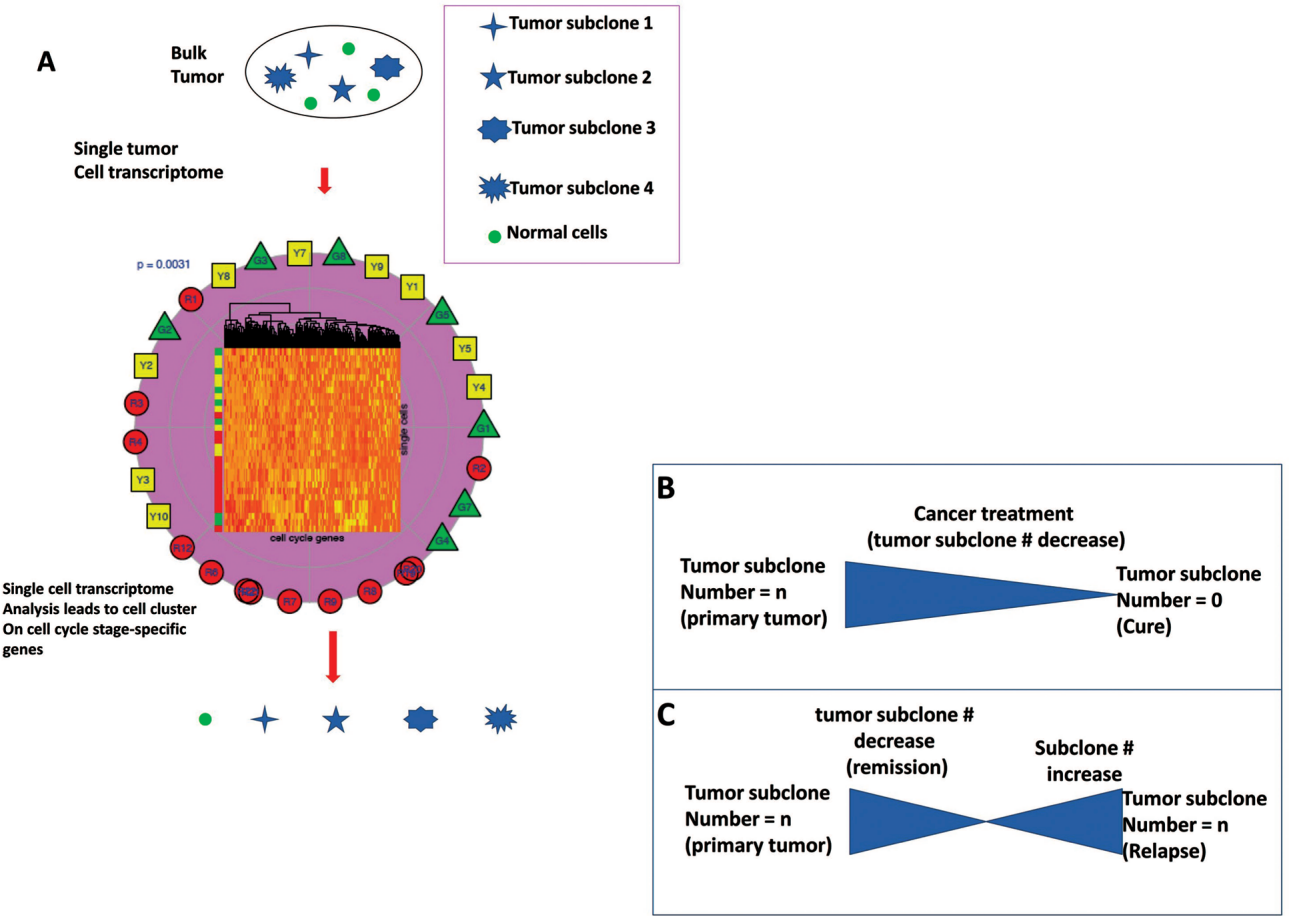
Sequential perturbations of cell-cycle-phase-specific genes derived from single-cell transcriptomes of patient tumors are applied to treatment (**A**) After organizing single-cell transcriptomes by similarity into a sequential order (center-clustering), expression levels of various cell-cycle-phase-specific genes were plotted to visualize the sequential perturbation of individual genes during the cell cycle, a virtual time series. Expression levels were scaled from 0 (undetectable) to 1 (maximum expression). Cell cycle phases were defined and colored. As expected, G0/G1-specific genes had higher expression levels in the G0/G1 phase and an S-specific gene was mainly expressed within the S phase. G2/M-specific genes had high expression levels in G2/M phase and early G0/G1 phase. The sequential expression order suggests that mRNAs of many G2/M-specific genes are not degraded until early in G0/G1 phase after cell division. (**B**, **C**) Cancer subclones are defined by single-cell transcriptome-clustered cell cycle gene clustering (all cells in a subclone share and stay at one cell-cycle stage, which is used to guide treatment. The effective therapeutics drive the number of tumor subclones decrease while the number of tumor subclones at relapse increase.

More specifically, the results of these single-cell transcriptomes provide details relating to define perturbation of critical genes during the cell cycle. Such concentrating on cell cycle related genes reveals a possible mechanism for the greatest prognostic power of MammaPrint^®^. Our results, which have identified an additional 23 cell cycle-related genes, bring the total number of predicative genes to 38, thereby significantly improving the clinical value of the MammaPrint^®^ panel. We propose that these results can be applied to focus potential strategies to create more efficacious, targeted combinations of drugs, specifically addressing the appropriate cells in the correct cell cycle stage [9, 10]. We suggest this cell cycle stage index (i.e., mapping out a spectrum of a specific phase of the cell cycle for a particular single cell) can be applied in cancer treatment (Figure 3) by monitoring subclonal growth through determination of switchboard signals (i.e., cytokines and chemokines) which may transform cells from dormancy to dominating subclones [11]. Clinical applications of cocktails of targeting a specific phase of the cell cycle can be applied to modulate the subclonal evolution for prognosis, thereby guiding the timing of therapeutic intervention. The 38-cycle-related genes in the MammaPrint^®^ 70-gene panel, as confirmed by clinical specimens (Tables 1, 2, 3), shed new light on subclonal evolution of common cancer treatment resistance, as certain cell-cycle stages, such G0, show resistance to treatment. This suggests that management of cell cycle stages can be therapeutic effective as it is tumor-subclone-specific, which is cell-cycle-dependent (Figure 3).

Such single-cell knowledge of categorizing distinct subpopulations of an organ may serve as a starting point to reconstruct the origins for the different subtypes of any organ-specific disease [12]. Single-cell identity may serve as a resource to map out the defined changes occurring during disease onset, potential to improve methods of detection and treatment. Next generation sequencing technology enables single-cell mRNA sequencing (scRNAseq) to create a high-resolution “molecular census of recognition” that was previously unseen cellular differences [13]. Isolated from prostate tumor and associated cells, for example, Calcinotto and colleagues identified two IL-23-regulated proteins (STAT3 and RORγ) that support androgen-receptor-associated tumor growth [14] in myeloid-derived suppressor cells (MDSCs) (including monocytes and neutrophils) at an immature state [15]. From insights into this immature state, novel therapies of either antibody (blocks IL-23) or enzalutamide (androgen-receptor inhibitor) were employed to shrink the tumors [16].

To secure efficacy of cell cycle-based therapy, physicians need to track down the spatiotemporal changes of each cell within breast tissue at molecular, cellular and tissue levels. Technologies (next generation sequencing technology, single-cell mRNA sequencing for single-cell gene signatures, and positioning imaging system for biomarkers [17]) can capture the spatiotemporal information that defines the cell-by-cell origins of breast cancer, in its earliest phases that acquire genetic alterations, potential for early cancer detection, leading to cancer prevention, slowing cancer progression or early management for co-survival with cancer [11], before it turns into a life-threatening cancer burden [18]. Such non-invasive imaging-based analysis of spatiotemporal gene-expression patterns [17] in single cells may shed new light onto the developmental processes that lead to “wait-and-watch” approaches [18], by defining “therapeutic windows” [19], with multiple check points of the full spectrum of cellular diversity that exists in the human body, in cancer initiation, progression and metastasis.

Cell cycle gene regulation plays a critical role not only in repair after tissue injury (e.g., stem cell-mediated regeneration), but also in cancer surveillance, and more recently has been employed as a tool in immunotherapy-based “wait-and-watch” approaches to cancer [18, 20]. Cell cycle gene regulation is tightly regulated by essential kinases such as members of the CDK family and their effectors. Monitoring cell cycle activity—including phenotyping immune cell and cancer stem cell subsets, tracking cell proliferation, and measuring cytokine production—can provide insights into the overall status of cancer in patients, and identify those cell populations which manage to survive cancer treatments. However, conventional gene expression profiling, which relies on population averaging of millions of heterogenous cells (malignant vs. normal cells), cannot realistically be expected to detect fine perturbations in gene expression that are precisely regulated on both temporal and spatial scales. Consequently, expression perturbations of many cell-cycle-related genes may remain masked in crude cell lysate analysis. The expression patterns of these cell-cycle genes as predicted by this algorithm remain to be validated in clinical trials for prognosis of other types of cancers.

Conclusion: With single-cell transcriptome analysis, we reveal that the previously unknown predictive power of MammaPrint^®^ signature panel arises from the cell cycle genes it profiles, and that the expression patterns of these cell cycle genes can be used for prognosis of other types of cancers.

## MATERIALS AND METHODS

### Cell culture and single-cell cDNA synthesis

H9 human embryonic stem cells (WA09, WiCell Research Institute, Inc.) were maintained with a feeder-free protocol as previously described [21, 22]. HeLa F. (RIKEN Cell Bank RCB2812) cells (Fucci cells) [23] were grown under standard conditions and cultured in Advanced DMEM (Invitrogen, Carlsbad, California), 1% Fetal Bovine Serum (HyClone, South Logan, Utah) and 0.5% penicillin-streptomycin (Invitrogen, Carlsbad, California). Cells were trypsinized using TrypLE (GIBCO) and resuspended in 1X phosphate-based buffer (PBS). Single-cell encapsulation was performed through hydrodynamic flow controlled by syringe pumps connected to a custom-fabricated microfluidic device as described previously [24]. The single-cell encapsulation, cDNA synthesis and amplifications in a nanoliter scale were carried out as previously reported [25, 26] Briefly, single cells were encapsulated into 600 pico-liter droplets with T-junction microfluidic devices or laser cavitation devices [27]. These droplets then were manipulated into 10 nano-liter reactors for reverse transcription reaction and diluted to 10 μl. The resulting products were then stored at −20°C until they were used for quantitative PCR or microarray gene expression profiling (Illumina). Samples were prepared for PCR amplification and subsequent microarray analysis by adding preamplification master mix (Illumina, San Diego, CA) as described previously [25]. For comparison, a Single-Cell Preamplification Kit (Life-Technologies, CA) was also used for cDNA synthesis and subsequently for qPCR verification.

### RNA-seq analysis

For RNA-seq samples, trimmed reads were mapped onto human genome hg38 using Tophat 2.0.8 as implemented in Flow with default settings, using Gencode 20 annotation (www.gencodegenes.org) as guidance. Gencode 20 annotation was used to quantify aligned reads to genes/transcripts using the Partek E/M method. [28] Read counts per gene in all samples were normalized using Upper Quartile normalization [29] and analyzed for differential expression using Partek's Gene Specific Analysis method (genes with less than 10 reads in any sample were excluded). To generate a list of significantly differentially expressed genes among different tissues of the same patient, a cutoff of FDR adjusted *p* < 0.05 (Poisson regression) and fold change >|2| was applied.

### Gene expression normalization and QC

Illumina gene expression data from each sample was processed and normalized independently of the other tissue/sample types. Analyses were restricted to genes significantly expressed in all single HeLa cells and lysates at a nominal *p*-value of 0.01, yielding 2,181 significant expression features out of 29,377 annotated probes on the array. For assessing agreement between single-cell data and lysate data, both were processed using a log 2 transformation followed by quantile normalization. To maximize the signal-to-noise ratio for fine mapping of single cells to the cell cycle, a more comprehensive approach was taken. The Lumi R package [30] was used to employ a variance-stabilizing transformation [31] that utilizes technical replicates on the array followed by Robust Spline Normalization. Extreme outliers across single cells were identified on a probe-by-probe basis and the values were set to ‘missing’ if they were more extreme than three interquartile ranges away from the first or third quartiles. However, no more than one outlier exceeded this criterion per probe. Missing data were then inputted using a nearest-neighbor averaging method.

### Statistical analysis

In cells that are approximately homogeneous with respect to factors other than their cell cycle stages, expression of cell cycle-related genes were used to determine the position in the cell cycle of each cell in relation to others. That is, similarity between cells in expression data was used to reflect similarity in cell cycle stage. We included in this analysis not only expressed genes (as described above), but also genes for which existing literature provided us with some evidence of their involvement in the cell cycle. Specifically, the inclusion criteria required that genes be classified in the GO category *cell cycle process* (GO:0022402). Among the many approaches that have been applied to clustering genes based on microarray expression patterns, Ray *et al*. [32] applied a traveling salesman problem (TSP) solver called Concorde [33] to obtain a one-dimensional ordering of genes within clusters. We used Concorde to estimate the shortest Hamiltonian path (a variation of the TSP where the path does not end at the starting position) based on Euclidean distance through the single cells (rather than genes). This approach required the construction of an adjacency matrix for single cells, which was generated using a network-based approach usually applied to the identification of co-expressed modules of genes [34].

The cell cycle status of individual cells forms a perturbation map, i.e., a diagram of how expression of a gene is perturbed as a cell passes through the cell cycle. This perturbation map was used with the Self-Organizing Maps (SOM) [35] approach to identify clusters of genes with the same perturbation signature. Genes with perturbation patterns similar to known cell cycle genes were inferred to be new candidate cell cycle genes.

### Oncogene profiling database

Multiple cancer profiling databases were used for cancer profile analyses, including Cancer Program Data Resources | Cancer Program Resource Gateway, ONCOMINE (Cancer Microarray Database and Integrated Data), Cancer RNA-Seq Nexus (database of phenotype-specific genome-wide transcriptome profiling of cancerous and normal tissue samples), Cancer Gene Expression Database (CGED) (database for gene expression profiling with accompanying clinical information of human cancer), Genomics of Drug Sensitivity in Cancer, Cancer atlas (The Human Protein Atlas), The COSMIC (Catalogue of Somatic Mutations in Cancer) database and website (Sanger Institute), Genomic Profiling in Cancer Patients (ClinicalTrials.gov), The Cancer Genome Atlas (TCGA), and International Cancer Genome Consortium. The relevant biomarkers were used to align with the single cell transcriptomes.

## Abbreviations

CGED: Cancer gene expression database
COSMIC: Catalogue of Somatic Mutations in Cancer
NEJM: New England Journal of Medicine
TCGA: The Cancer Genome Atlas
